# Subjective Cognitive Decline Among Adults Aged ≥45 Years — United States, 2015–2016

**DOI:** 10.15585/mmwr.mm6727a1

**Published:** 2018-07-13

**Authors:** Christopher A. Taylor, Erin D. Bouldin, Lisa C. McGuire

**Affiliations:** ^1^Division of Population Health, National Center for Chronic Disease Prevention and Health Promotion, CDC; ^2^Department of Health and Exercise Science, Beaver College of Health Sciences, Appalachian State University, Boone, North Carolina.

Subjective cognitive decline (SCD) is the self-reported experience of worsening or more frequent confusion or memory loss within the previous 12 months (*1*,*2*) and one of the earliest noticeable symptoms of Alzheimer’s disease (Alzheimer’s), a fatal form of dementia (i.e., a decline in mental abilities severe enough to interfere with everyday life) (*1*). Alzheimer’s is the most common form of dementia, although not all memory loss results from Alzheimer’s (*3*). To examine SCD, CDC analyzed combined data from the 2015 and 2016 Behavioral Risk Factor Surveillance System (BRFSS) surveys. Overall, 11.2% of adults aged ≥45 years reported having SCD, 50.6% of whom reported SCD-related functional limitations. Among persons living alone aged ≥45 years, 13.8% reported SCD; among persons with any chronic disease, 15.2% reported SCD. Adults should discuss confusion or memory loss with a health care professional who can assess cognitive decline and address possible treatments and issues related to chronic disease management, medical care, and caregiving.

BRFSS is a state-based, random-digit–dialed telephone survey of noninstitutionalized adults aged ≥18 years in all 50 states, the District of Columbia (DC), and several U.S. territories.* The six-question cognitive decline module (optional for states in 2015 and 2016) examines how SCD affects the life of respondents aged ≥45 years, including difficulties performing activities or caring for themselves. Overall, 49 states (all except Pennsylvania), Puerto Rico, and DC administered the module in one or both years. For five states that administered the module in both years, only 2016 data were included in this analysis. For the BRFSS surveys in 2015 and 2016, the overall combined landline and cellular telephone response rates among states, Puerto Rico, and DC ranged from 30.7% to 65.0% (median = 47.1%).^†^

Respondents who answered affirmatively to the question “During the past 12 months, have you experienced confusion or memory loss that is happening more often or is getting worse?” were classified as having SCD. Respondents with SCD were asked if SCD caused them to give up day-to-day activities such as cooking, cleaning, taking medications, driving, or paying bills; how often they needed and could receive necessary assistance with those activities; how often SCD interfered with their ability to work, volunteer, or engage in social activities; and whether they had discussed SCD with a health care professional. Respondents who reported “always,” “usually,” or “sometimes” (as opposed to “rarely” or “never”) giving up day-to-day activities or interference with ability to work, volunteer, or engage in social activities were classified as having SCD-related functional limitations.

Data were examined by age group, sex, race/ethnicity, education level, veteran status, employment, and living alone. Chronic disease status was ascertained by history of heart disease; stroke, or cerebrovascular disease; asthma; lung disease; cancer (other than skin); arthritis; or diabetes. Data were analyzed using statistical software and methods that accounted for the complex survey design and weighted data. Prevalence rates were unadjusted.

Among adults aged ≥45 years, 11.2% reported SCD, 50.6% of whom reported SCD-related functional difficulties (Table 1). SCD prevalence increased with age, from 10.4% among adults aged 45–54 years to 14.3% among those aged ≥75 years and was lower among college graduates (7.0%) than among those with less than high school education (18.2%). The prevalence of SCD-related functional difficulties among college graduates (30.8%) was half that of those without a high school diploma (64.9%). Among persons living alone, 13.8% reported SCD; 55.7% of those reported SCD-related functional difficulties (Table 1).

**TABLE 1:** Characteristics of adults aged ≥45 years who reported subjective cognitive decline (SCD) and associated functional limitations — Behavioral Risk Factor Surveillance System, 49 states,* Puerto Rico, and the District of Columbia, 2015–2016

Characteristic	SCD		Functional limitations among those reporting SCD	
	No. of respondents^†^	% (95% CI)^§^	No. of respondents^†^	% (95% CI)^§^
**Overall**	**227,393**	**11.2 (10.8–11.5)**	**23,705**	**50.6 (49.0–52.2)**
**Age group (yrs)**				
45–54	48,563	10.4 (9.7–11.1)	4,868	59.8 (56.4–63.2)
55–64	68,835	11.4 (10.8–12.0)	7,081	56.9 (54.3–59.6)
65–74	64,472	9.9 (9.3–10.5)	5,978	39.3 (36.4–42.3)
≥75	45,523	14.3 (13.3–15.2)	5,778	37.5 (34.1–41.0)
**Sex**				
Men	92,639	11.4 (10.8–11.9)	10,095	47.6 (45.3–49.9)
Women	134,743	11.0 (10.6–11.5)	13,609	53.2 (51.0–55.5)
**Race/Ethnicity^¶^**				
White	184,742	10.8 (10.5–11.2)	18,622	44.9 (43.2–46.7)
Black	16,370	13.2 (12.0–14.3)	1,991	64.4 (59.5–69.4)
American Indian/Alaska Native	3,232	19.6 (16.0–23.2)	498	73.4 (64.8–82.1)
Asian or Native Hawaiian/Other Pacific Islander	3,223	6.8 (4.3–9.3)	261	39.7 (23.9–55.5)
Other race or multiracial	4,681	15.4 (12.6–18.2)	664	55.9 (46.0–65.8)
Hispanic	11,680	11.2 (9.8–12.7)	1,267	65.8 (58.8–72.8)
**Highest education level**				
Less than a high school diploma	17,602	18.2 (16.8–19.5)	3,110	64.9 (60.6–69.1)
High school diploma	65,474	11.6 (11.0–12.1)	7,415	53.2 (50.6–55.8)
Some college	61,574	11.5 (10.8–12.2)	6,826	49.1 (46.0–52.1)
College graduate	82,094	7.0 (6.5–7.5)	6,290	30.8 (27.7–33.8)
**Veteran status**				
Veteran	35,738	13.6 (12.7–14.5)	4,611	42.5 (39.0–54.1)
Not a veteran	191,434	10.8 (10.4–11.1)	19,065	52.4 (50.6–54.1)
**Employment status**				
Employed/Self-employed	91,486	6.0 (5.7–6.4)	5,209	31.1 (28.2–33.9)
Unemployed	7,184	16.9 (14.5–19.3)	1,109	60.0 (51.5–68.5)
Homemaker	12,313	8.4 (6.9–10.0)	1,057	45.7 (36.7–54.7)
Student	431	5.8 (2.9–8.6)	40	76.3 (61.0–91.5)
Retired	94,918	11.3 (10.8–11.9)	9,934	38.2 (35.7–40.7)
Unable to work	19,832	34.8 (33.1–36.5)	6,221	79.4 (77.1–81.7)
**Household status**				
Lives alone	78,274	13.8 (13.2–14.4)	9,640	55.7 (53.3–58.0)
Does not live alone	148,038	10.4 (9.9–10.8)	13,957	48.2 (46.2–50.2)
**Any chronic disease**				
Yes	143,954	15.2 (14.7–15.7)	19,589	53.9 (52.2–55.6)
No	83,381	5.2 (4.8–5.7)	4,103	36.1 (32.0–40.1)

**Abbreviation:** CI = confidence interval.

* Includes all states except Pennsylvania.

^†^ Unweighted sample of respondents. Categories might not sum to the sample total because of missing responses.

^§^ Weighted percentage and 95% CI.

^¶^ All persons who reported a racial group were non-Hispanic. Those who reported Hispanic ethnicity might be members of any racial group.

The prevalence of SCD varied by state (Table 2). The lowest prevalence of SCD was reported in South Dakota (6.0%), and the highest was reported in Nevada (16.3%).

**TABLE 2:** Reported subjective cognitive decline (SCD) among adults aged ≥45 years, by state — Behavioral Risk Factor Surveillance System, 49 states,* Puerto Rico, and the District of Columbia, 2015–2016

State	No. of respondents^†^	% (95% CI)^§^
**Overall**	**254,821**	**11.2 (10.8–11.5)**
Alabama	5,811	12.9 (11.7–14.1)
Alaska	2,044	11.3 (9.3–13.4)
Arizona	6,188	13.4 (12.1–14.8)
Arkansas	4,347	16.2 (14.2–18.2)
California	2,268	11.7 (9.7–13.8)
Colorado	4,764	10.8 (9.5–12.1)
Connecticut	4,305	7.3 (6.1–8.5)
Delaware	2,914	8.8 (7.4–10.2)
District of Columbia	3,185	12.1 (9.5–14.7)
Florida	3,555	11.3 (9.9–12.7)
Georgia	3,487	14.0 (12.4–15.7)
Hawaii	5,007	8.9 (7.8–10.0)
Idaho	3,934	10.8 (9.4–12.1)
Illinois	3,773	9.6 (8.4–10.9)
Indiana	8,689	10.5 (9.6–11.4)
Iowa	4,776	9.3 (8.2–10.4)
Kansas	4,442	9.1 (8.0–10.2)
Kentucky	7,419	12.1 (10.9–13.2)
Louisiana	3,433	14.6 (12.9–16.2)
Maine	4,676	10.3 (9.0–11.5)
Maryland	5,074	10.6 (8.8–12.5)
Massachusetts	5,916	9.3 (8.1–10.4)
Michigan	2,070	12.1 (10.2–13.9)
Minnesota	11,798	8.7 (8.0–9.3)
Mississippi	4,684	12.9 (11.5–14.4)
Missouri	5,456	10.4 (9.1–11.8)
Montana	4,473	9.8 (8.6–11.1)
Nebraska	6,405	9.4 (8.3–10.5)
Nevada	2,142	16.3 (13.3–19.4)
New Hampshire	5,125	8.9 (7.8–9.9)
New Jersey	5,637	9.1 (7.9–10.4)
New Mexico	4,507	12.5 (10.9–14.0)
New York	8,353	10.3 (8.9–11.8)
North Carolina	4,296	10.7 (9.5–11.9)
North Dakota	3,675	9.9 (8.6–11.3)
Ohio	9,464	10.7 (9.6–11.8)
Oklahoma	2,626	13.6 (11.6–15.6)
Oregon	3,675	11.3 (10.0–12.6)
Rhode Island	4,835	11.5 (10.0–12.9)
South Carolina	8,683	12.1 (11.1–13.1)
South Dakota	5,407	6.0 (4.8–7.1)
Tennessee	4,538	13.6 (12.2–15.1)
Texas	5,185	13.1 (11.3–14.9)
Utah	3,428	9.6 (8.3–10.9)
Vermont	4,991	9.8 (8.6–11.0)
Virginia	6,172	8.9 (8.0–9.8)
Washington	10,356	11.1 (10.3–11.9)
West Virginia	4,231	10 (8.9–11.1)
Wisconsin	4,512	10.9 (9.4–12.3)
Wyoming	4,438	11.2 (9.7–12.7)
Puerto Rico	3,652	6.6 (5.6–7.6)

**Abbreviation:** CI = confidence interval.

* Includes all states except Pennsylvania.

^†^ Unweighted sample of respondents. Categories might not sum to the sample total because of missing responses.

^§^ Weighted percentage and 95% CI.

Nearly twice the percentage of persons reporting SCD-related functional limitations had talked to a health care professional (58.1%) compared with those without functional limitations (30.4%) (Table 3). Among persons with a functional difficulty, 81.1% reported having given up household activities or chores because of SCD, and 73.3% reported that SCD interfered with their ability to work, volunteer, or engage in social activities.

**TABLE 3:** Percentage of adults aged ≥45 years with subjective cognitive decline (SCD), by SCD-related functional limitation status in preceding 12 months — Behavioral Risk Factor Surveillance System, 49 states,* Puerto Rico, and the District of Columbia, 2015–2016

Characteristic	All with SCD		With SCD and functional limitations		With SCD but no functional limitations	
	Unweighted no.	% (95% CI)	Unweighted no.	% (95% CI)	Unweighted no.	% (95% CI)
Ever discussed SCD with a health care professional	23,853	45.4 (43.8–46.9)	11,111	58.1 (55.9–60.3)	12,398	30.4 (34.6–35.6)
Gave up household activities or chores because of SCD^†^	23,682	40.4 (38.9–42.0)	11,078	81.1 (79.0–83.1)	12,456	—^§^
SCD interfered with ability to work, volunteer, or engage in social activities outside the home^†^	23,675	36.5 (35.0–38.1)	11,049	73.3 (71.4–75.3)	12,456	—^§^

**Abbreviation:** CI = confidence interval.

* Includes all states except Pennsylvania.

^†^ Always, usually, or sometimes.

^§^ By definition.

## Discussion

SCD can be a symptom of early-stage dementia conditions, including Alzheimer’s (*1*,*2*). Not everyone who reports SCD will develop dementia, but some studies have shown that half of older adults with subjective memory complaints go on to develop more severe cognitive decline within 7–18 years (*1*,*4*,*5*). Even without progression to more severe cognitive impairment, SCD might signify a decreased ability for self-care. Inability to perform activities important to daily living such as preparing meals or managing money affect the ability to live independently and might also affect the ability to socialize or remain fully employed.

These findings are similar to those from an analysis of persons aged ≥60 years in 21 states from the 2011 BRFSS survey, which found a 12.7% prevalence of SCD (*6*). In that study, the highest prevalence was among Hispanics (16.9%) and the lowest was among non-Hispanic blacks (11.8%), in contrast to the current study, which found the highest prevalence among non-Hispanic American Indians and Alaska Natives (19.6%) and the lowest among non-Hispanic Asians or Native Hawaiians/Other Pacific Islanders (6.8%). The inclusion of additional states and the expansion of the age groups might have contributed to these differences.

In both 2011 (*6*) and 2015–2016, a higher SCD prevalence was found among adults aged ≥75 years than among those aged 45–74 years. This is similar to the prevalence of Alzheimer’s, according to 2018 data from the Alzheimer’s Association, which found an estimated 3% of persons aged 65–74 years, 17% of persons aged 75–84 years, and 32% of persons aged ≥85 years had Alzheimer’s (*1*,*7*). This analysis found a higher prevalence of SCD and related functional limitations in persons with less formal education, similar to previously reported patterns of higher dementia prevalence in persons with less formal education (*8*).

Younger adults might be more likely to attribute limitations in their lifestyle to SCD or might be more sensitive to its effects. Conversely, older adults might be less aware of the effects of SCD or consider it a normal part of aging. Among persons aged 45–54 years, 10.4% reported SCD, and 59.8% of those persons reported SCD-related limitations that affected work, household chores, or social activities. Although Alzheimer’s is rare in persons aged <65 years, the finding of SCD and related functional limitations among younger adults could indicate early symptoms of cognitive decline that can be a precursor to memory disorders and dementia like Alzheimer’s. These functional limitations might have important health and economic impacts. Adults aged 45–54 years are in their prime working years, when salaries peak, workers are most productive, and when workers contribute to their retirements and consume goods and services (*9*). An inability to work during these years might have financial implications for these adults and their families. Persons with SCD-related functional limitations might have to reduce their time working or leave the workforce entirely; in this study, nearly three fourths of those with a functional difficulty reported that SCD interfered with their ability to engage in activities outside the home, including working.

Fewer than half (45.4%) of respondents with SCD reported speaking to a health care professional about it. More than half of those with SCD-related functional limitations reported speaking to a health care professional about SCD compared with fewer than one third of persons without such limitations, suggesting that limitations in ability to perform instrumental activities of daily living might prompt discussion with a health care professional. Persons might incorrectly believe that cognitive decline is an inevitable part of aging, which could discourage them from consulting a health care professional. CDC encourages persons with confusion or memory loss to talk to a health care professional. After evaluation, even if treatment of symptoms is not an option, early assessment of cognitive issues can facilitate addressing potential safety issues, discussion of advanced care planning, including the need for caregiving, and ensuring receipt of appropriate information and referrals (*10*). Early assessment is important because memory issues can affect a person’s ability to manage their health; among those reporting other chronic health conditions, 15.2% also had SCD.

The findings in this report are subject to at least three limitations. First, data on SCD are self-reported. Whereas the SCD module was cognitively tested, it is not administered alongside an objective measure of cognitive performance. Therefore, the accuracy of the reports of SCD is unknown. Second, response bias might affect response to SCD questions and might underestimate SCD prevalence. Finally, BRFSS is not administered to persons with known cognitive problems who might not generate reliable data. In addition, BRFSS is only administered to noninstitutionalized adults, excluding adults living in long-term care facilities, where a proportion of residents have SCD. Therefore, these results cannot be used to estimate the prevalence of SCD across all U.S. populations.

Cognitive decline is an important public health issue affecting older adults, their families, and their caregivers, as well as the economy and health care system. As a precursor to dementia, including Alzheimer’s, SCD can impair a person’s ability to care for themselves by limiting their ability to work, particularly those adults who report SCD in their prime working years (i.e., 45–54 years). Estimating the prevalence of SCD might allow states to plan for those who might develop dementia in the future.

**Summary**

**What is already known about this topic?**

Subjective cognitive decline (SCD) is a form of impairment in which more frequent or worsening confusion or memory loss can affect the ability to care for oneself.

**What is added by this report?**

Among adults aged ≥45 years, 11.2% reported SCD, including 10.4% of adults aged 45–54 years. Among all persons who reported SCD, only 45.4% had discussed it with a health care professional.

**What are the implications for public health practice?**

Adults with confusion or memory loss should talk to a health care professional who can assess cognitive decline and address possible treatment of symptoms, management of other co-occurring chronic health conditions, advance care planning, and caregiving needs, and who ensures that the patient receives appropriate information and referrals.

## Footnotes

* https://www.cdc.gov/brfss.

† https://www.cdc.gov/brfss/annual_data/2015/pdf/2015-sdqr.pdf and https://www.cdc.gov/brfss/annual_data/2016/pdf/2016-sdqr.pdf.
